# Adjuvant Radiotherapy for Intermediate-Risk Early-Stage Cervical Cancer Post Radical Hysterectomy: A Systematic Review and Meta-Analysis

**DOI:** 10.3390/jcm14114002

**Published:** 2025-06-05

**Authors:** Pedro Henrique Costa Matos da Silva, Gabriela Oliveira Gonçalves Molino, Maírla Marina Ferreira Dias, Ana Gabriela Alves Pereira, Nicole dos Santos Pimenta, Deivyd Vieira Silva Cavalcante, Ana Clara Felix de Farias Santos, Sarah Hasimyan Ferreira, Rodrigo da Silva Santos, Angela Adamski da Silva Reis

**Affiliations:** 1 Department of Obstetrics and Gynecology, Federal University of Goiás, Goiânia 74690-900, GO, Brazil; rdssantos@ufg.br; 2 Gynecologic Oncology Service, Federal District Base Hospital, Brasília 70040-010, DF, Brazil; 3 Department of Medicine, Federal University of Health Sciences of Porto Alegre, Porto Alegre 90050-170, RS, Brazil; ogm.gabi@gmail.com; 4 Department of Medicine, Federal University of Campina Grande, Campina Grande 58429-900, PB, Brazil; mairlafd@gmail.com; 5 Department of Medicine, State University of São Paulo, São Paulo 05508-220, SP, Brazil; aga.pereira@unesp.br; 6 Department of Medicine, Federal University of the State of Rio de Janeiro, Rio de Janeiro 22290-240, RJ, Brazil; nnicolepimenta@gmail.com; 7 Department of Pharmacy, Federal University of Maranhão, São Luís 65080-805, MA, Brazil; deivydcavalcante@my.unthsc.edu; 8 Department of Pharmacy, City University of São Paulo, São Paulo 05305-000, SP, Brazil; anafelix625@gmail.com; 9 Departamento de Ginecologia e Obstetrícia, Faculdade de Medicina, Universidade Federal de Goiás, Goiânia 74690-900, GO, Brazil; sarah.hasimyan@gmail.com

**Keywords:** cervical cancer, intermediate risk, *Sedlis* criteria, radiotherapy, recurrence

## Abstract

**Background:** The risk of recurrence of early-stage cervical cancer (CC) is associated with prognostic factors such as tumor size, lymphovascular space invasion (LVSI), and deep stromal invasion (DSI). However, the adjuvant pelvic radiotherapy (RT) following surgery to reduce the risk of recurrence in “intermediate risk” remains controversial. This study aims to evaluate the role of adjuvant RT in the recurrence and identify prognostic factors. **Methods:** A systematic search of PubMed, Embase, and Cochrane databases was performed to identify studies comparing adjuvant RT versus no adjuvant treatment in early-stage CC patients with intermediate-risk factors defined by GOG-92 criteria. Outcomes were recurrence, local recurrence, death, 5-year overall survival (5y-OS), and 5-year disease-free survival (5y-DFS). Tumor size ≥ 4 cm, LVSI, and DSI were also evaluated as prognostic factors for recurrence. Statistical analysis was performed using Review Manager 7.2.0. Heterogeneity was assessed with I^2^ statistics. **Results:** A total of 1504 patients from nine studies were included; only one study was a randomized controlled trial, while the others were retrospective cohorts. Adjuvant RT was used to treat 781 patients (52%). Median follow-up ranged from 48 to 120 months. Recurrence (OR 0.75; 95% CI 0.38–1.46; *p* = 0.39), local recurrence (OR 0.73; 95% CI 0.44–1.20; *p* = 0.22), death (OR 0.97; 95% CI 0.52–1.80; *p* = 0.91), 5y-OS (OR 1.22; 95% CI 0.36–4.18; *p* = 0.75), and 5y-DFS (OR 0.78; 95% CI 0.42–1.43 *p* = 0.42) revealed no statistically significant differences between adjuvant RT and observation groups. TS ≥ 4 cm was an independent prognostic risk factor for recurrence (HR 1.83; 95% CI 1.12–2.97; *p* = 0.02). **Conclusions:** Our findings suggest that adjuvant RT does not reduce recurrence risk in early-stage cervical cancer. Consider TS ≥ 4 cm as a significant prognostic factor for recurrence. Adjuvant RT in intermediate-risk patients should be considered with caution due the lack of significant improvement in recurrence until the CERVANTES and GOG-0263 trial results become available.

## 1. Introduction

Cervical cancer (CC), despite being a preventable disease, remains a significant global public health challenge. Even with the World Health Organization’s (WHO) *Global Strategy to Accelerate the Elimination of Cervical Cancer*, CC was the fourth most common cancer among females, accounting for an estimated 661,021 new cases and 348,189 deaths worldwide in 2022 [1]. The incidence and mortality rates are clearly associated with the Human Development Index (HDI). In low-HDI countries, CC is the second most common type of cancer and the third most common cause of cancer mortality, where incidence and mortality rates are approximately three and six times higher, respectively, compared to countries with a high HDI [1,2].

CC staging has changed significantly over time and is currently based on the staging system of the Federation of Gynecology and Obstetrics (FIGO) 2018 [3,4]. In early-stage CC, which is limited to the cervix, primary surgery is preferred over primary radiation therapy (RT) due to comparable efficacy and long-term morbidity associated with RT, including some concerns related to sexual quality of life [5] and ovarian failure. However, RT with brachytherapy and concurrent platinum-containing chemotherapy are preferred therapies for locally advanced disease and tumor size > 4 cm (stages IB3 and IIA2) as suggested by the *National Comprehensive Cancer Network* (NCCN) [6].

Despite undergoing curative therapy, some patients may have recurrence. For those undergoing definitive surgery, the primary site of disease recurrence is local. The risk of recurrence is associated with prognostic factors, including tumor size, lymph node involvement, lymphovascular space invasion (LVSI), deep stromal invasion (DSI), and tumor histology [7,8,9]. The *Gynecologic Oncology Group* (GOG) randomized trial #92 (GOG-92) suggested that adjuvant pelvic RT following radical surgery significantly reduces the risk of recurrence in women with limited cervix disease who are classified as “intermediate risk”. This classification was defined as limited cervical disease combined with the following risk factors: DSI, LVSI, and tumor size ≥ 4 cm (Table S1) [7,8].

However, subsequent non-randomized studies [10,11] have shown enough local control in the group of patients after radical surgery without additional treatment, suggesting that observation might be equally effective. Furthermore, improvements in surgical techniques and more effective diagnostic exams may change the prognosis of these patients. Therefore, the role of adjuvant RT may be considered controversial and is supported by a single randomized clinical trial (RCT) performed more than 20 years ago [8]. Conservative treatments have been considered for CC, mainly due to the morbidities associated with RT.

This study aims to identify potential prognostic factors and evaluate the role of adjuvant radiotherapy in the recurrence of cervical cancer among patients with a history of surgical treatment and defined intermediate-risk factors.

## 2. Materials and Methods

This systematic review and meta-analysis were registered on the International Prospective Register of Systematic Reviews (PROSPERO) with registration number CRD42024587124 [12]. This study was designed in accordance with the *Preferred Reporting Items for Systematic Reviews and Meta-Analysis* (PRISMA) reporting guidelines [13].

### 2.1. Study Eligibility

The reports identified through the searches were screened for relevance, and those considered potentially eligible were reviewed based on the inclusion and exclusion criteria. Inclusion criteria for potentially relevant reports were RCTs or non-randomized cohort studies in which patients were selected to receive adjuvant RT versus no adjuvant therapy and studies involving patients with “intermediate-risk factors”, primarily treated with radical surgery and without compromised lymph nodes, surgical margins, or parametria. The definition of intermediate risk was based on the presence of at least two risk factors (tumor size ≥ 4 cm, LVSI, and DSI], as defined by Rotman et al. [8].

The inclusion of both randomized and non-randomized studies was considered to maximize the scope and relevance of the results. Non-randomized studies can introduce biases due to the lack of random control but also provide valuable insights, especially with the lack of randomized studies.

The exclusion criteria were reports involving patients with advanced-stage cervical cancer without an intervention or control group; reports with radiotherapy as primary treatment (i.e., radiotherapy before surgery); and reports lacking outcomes of interest. Data from patients who received chemotherapy were excluded from the analysis to ensure the homogeneity of the intervention. Studies with overlapping populations and those not matching the desired study design were also excluded. References were examined at the title/abstract level independently by two investigators (G.M., M.D.) and, if potentially suitable for inclusion, were retrieved as complete articles. Disagreements were resolved through discussion with a third reviewer (P.S.) until consensus.

### 2.2. Search Strategy

A systematic search was independently performed in the PubMed, Embase, and Cochrane Central Register of Controlled Trials databases by two investigators (G.M., M.D.) in August 2024. The full search strategy aimed to include any randomized trials or non-randomized cohort studies involving adjuvant radiotherapy treatment in patients with early-stage cervical cancer (CC) who were treated with radical hysterectomy (RH) and pelvic lymph node dissection and had intermediate risk factors as defined by the GOG-92 [7,8]. The full search details are available in the Supplementary Materials (Table S2). No language restriction was imposed. Additionally, manual searches were performed from the reference list of eligible primary reports and relevant review articles.

### 2.3. Data Extraction

Data from the chosen articles were extracted independently by two reviewers (A.P., N.P.) using an extraction form. The primary outcome of our analysis was recurrence. Secondary outcomes were death, 5 years of overall survival (5y-OS), and 5 years of disease-free survival (5y-DFS). A leave-one-out analysis was performed to evaluate the impact of individual studies on each of the outcomes. The study characteristics authors, year of publication, country, study design, sample size, mean follow-up, and patient characteristics were also recorded. Tumor size ≥ 4 cm (TS ≥ 4 cm), lymphovascular space invasion (LVSI), and deep stromal invasion (DSI) were also evaluated as prognostic factors for recurrence.

### 2.4. Quality and Evidence Assessment

Risk of bias was assessed by two researchers (M.D., P.S.) using the Cochrane Risk of Bias tool: Risk of Bias 2 (RoB 2) [14] for RCTs; and Risk of Bias In Non-randomized Studies of Interventions (ROBINS-I) [15]. Accordingly, “high-risk” bias was assigned to studies presenting a high risk of bias on any domain of the RoB 2 tool or some concerns for multiple domains, “some concerns” was assigned to studies presenting some concerns on any domain, and “low risk” of bias was assigned if otherwise. The layout was generated using Risk Of Bias VISualization (ROBIVS) [16]. Publication bias was assessed by visually inspecting funnel plots.

The overall quality of evidence was analyzed according to the Grading of Recommendation, Assessment, Development, and Evaluations (GRADE) guidelines [17]. The studies were labeled with very-low-, low-, moderate-, or high-quality evidence based on the presence of risk of bias, inconsistency of results, imprecision, publication bias, and magnitude of treatment effects. Due to the low number of included studies, neither publication bias nor a dose/response effect could be assessed.

### 2.5. Statistical Analysis

The treatment effects for binary endpoints were compared using Odds Ratios (ORs), with 95% confidence intervals (CIs), and Hazard Ratios (HRs) with 95% confidence intervals. Heterogeneity was assessed with the Cochrane Q-test, I^2^ statistics, and Tau-square using the restricted maximum-likelihood estimator [18]. *p*-Values > 0.10 and I^2^ values > 25% were considered significant for heterogeneity [19]. A leave-one-out analysis was performed to explore possible causes of heterogeneity among study results. Random-effect models were used for all outcomes. Data handling and conversion followed the guidelines of the Cochrane Handbook for Systematic Reviews of Interventions [20]. Meta-analyses were analyzed using Review Manager (RevMan) Version 7.2.0.

## 3. Results

### 3.1. Study Selection and Baseline Characteristics

**Figure 1:**
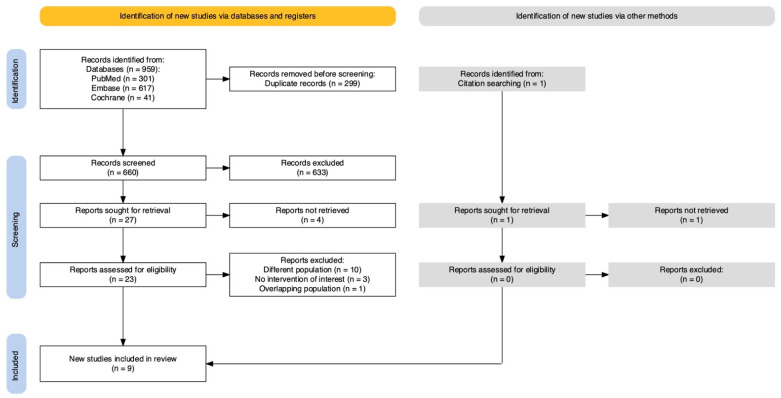
PRISMA flow diagram of study screening and selection.

The primary search resulted in 959 articles. After removing 299 duplicate reports and excluding 633 articles by title or abstract, 27 articles were sought for retrieval and 23 reports were assed for eligibility. After full-text evaluation, nine articles were included (Figure 1). Among these, eight were retrospective cohort studies (RCSs) and one was a randomized clinical trial (RCT). In total, 1504 patients were included, with 781 (52%) patients receiving radiotherapy adjuvant and 723 (48%) receiving standard treatment. The publication years ranged from 2006 to 2023. Median follow-up ranged from 48 to 120 months. Characteristics of the included trials and studies are presented in Table 1.

### 3.2. Pooled Analysis of All Studies

**Figure 2:**
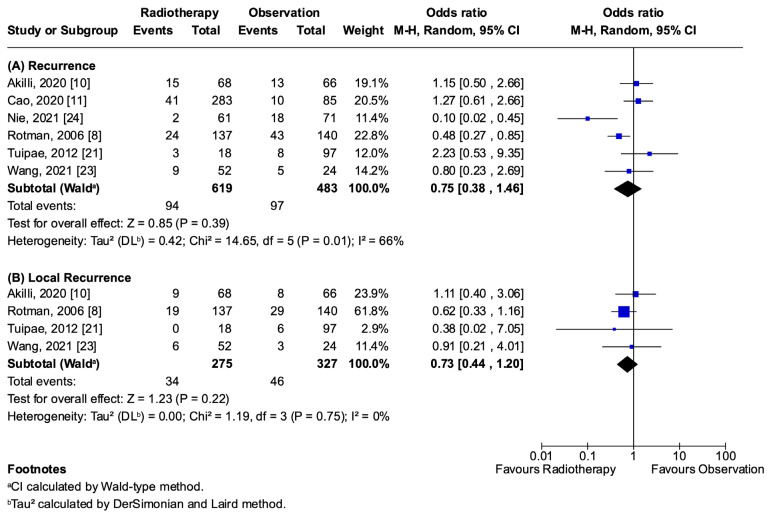
Recurrence forest plot: (**A**) recurrence [8,10,11,21,23,24]; (**B**) local recurrence [8,10,21,23].

**Figure 3:**
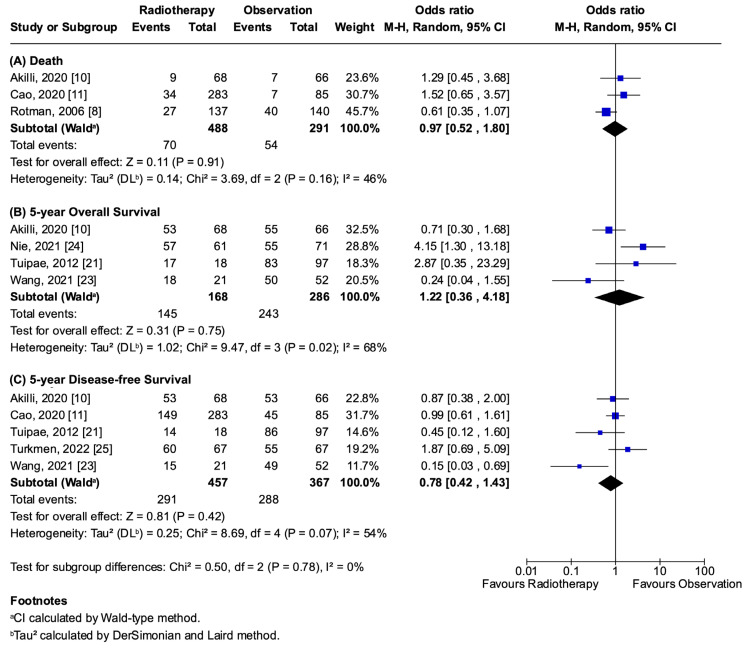
Secondary outcomes forest plot: (**A**) death [8,10,11]; (**B**) 5-year overall survival [10,21,23,24]; (**C**) 5-year disease-free survival [10,11,21,23,25].

The primary outcomes, recurrence (OR 0.75; 95% CI 0.38–1.46; *p* = 0.39; I^2^ = 66%; Figure 2A) and local recurrence (OR 0.73; 95% CI 0.44–1.20; *p* = 0.22; I^2^ = 0%; Figure 2B), showed no statistically significant difference between the adjuvant radiotherapy and observation groups. Similarly, the secondary outcomes, which included death (OR 0.97; 95% CI 0.52–1.80; *p* = 0.91; I^2^ = 46%), 5y-OS (OR 1.22; 95% CI 0.36–4.18; *p* = 0.75; I^2^ = 68%), and 5y-DFS (OR 0.78; 95% CI 0.42–1.43 *p* = 0.42; I^2^ = 54%), revealed no statistically significant differences (Figure 3). A comprehensive overview of the pooled analyses for each outcome is presented in Table S3.

A leave-one-out sensitivity analysis was performed for recurrence, local recurrence, death, 5y-OS, and 5y-DFS. Overall, no change was observed in the statistical significance of the outcome in each of the leave-one-out tests. However, heterogeneity changed for several outcomes. Changes in heterogeneity were noted when removing Nie et al. [24] in the outcome of recurrence, with a 66% to 45% reduction, although high heterogeneity remained. Similarly, when Cao et al. [11] were removed, we noticed a decrease in heterogeneity from 46 to 32% in the outcome of death. We observed a decrease in heterogeneity from 68 to 33% omitting Nie et al. [24] for the 5y-OS outcome and 54 to 4% omitting Wang et al. [23] for the 5y-DFS.

The observed decreases in heterogeneity likely stem from differences in methodology, population characteristics, and treatment protocols. Variations in study design, sample size, and statistical method (such as differing inclusion criteria, endpoints, and models) contribute significantly. Additionally, demographic factors like age, cancer stage, histological type, and risk factors introduce variability. Differences in treatment regimens, including the type and dosage of adjuvant therapy, also affect outcomes. Therefore, inconsistencies in data interpretation and reporting, such as varying definitions of some outcomes, further contribute to heterogeneity.

**Figure 4:**
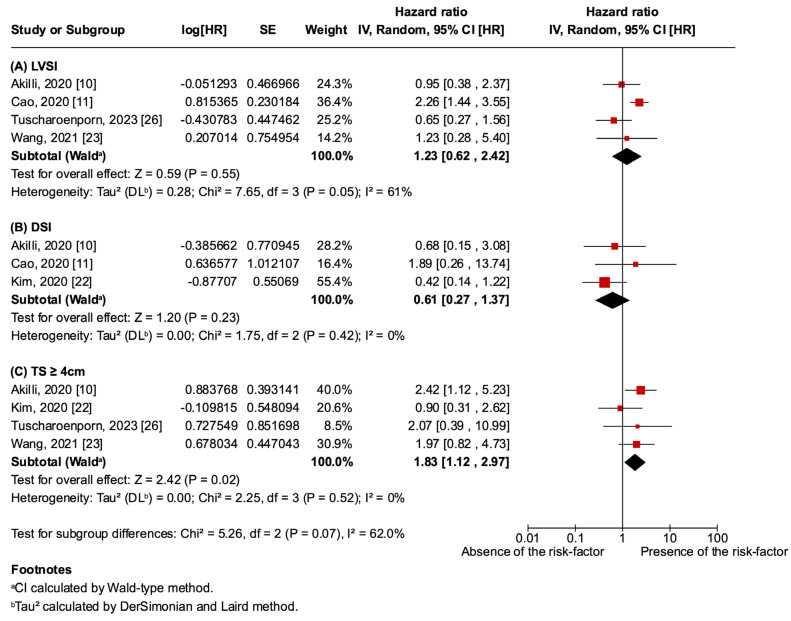
Prognostic risk-factor forest plot: (**A**) lymphovascular space invasion (LVSI) [10,11,23,26]; (**B**) deep stromal invasion (DSI) [10,11,22]; (**C**) tumor size (TS) ≥ 4 cm [10,22,23,26].

TS ≥ 4 cm, LVSI, and DSI were evaluated as prognostic risk factors for recurrence (Figure 4). LVSI (HR 1.23; 95% CI 0.62–2.42; *p* = 0.055; I^2^ = 61%) and DSI (HR 0.61; 95% CI 0.27–1.37; *p* = 0.23; I^2^ = 0%) showed no statistically significant difference between the presence or absence of the risk factors. However, TS ≥ 4 cm was an independent negative prognostic risk factor for recurrence (HR 1.83; 95% CI 1.12–2.97; *p* = 0.02; I^2^ = 0%). The analysis of combined risk factors was not conducted because of insufficient studies providing these data, preventing a comprehensive analysis of the interactions between risk factors.

### 3.3. Quality and Evidence Assessment

**Figure 5:**
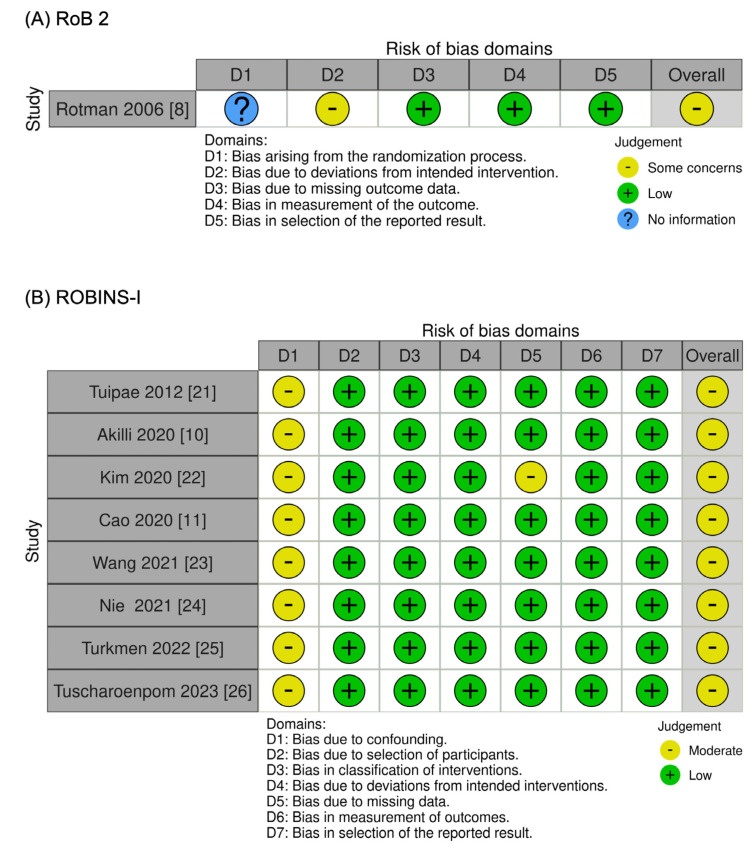
Cochrane Risk of Bias tool: (**A**) RoB 2 [8]; (**B**) ROBINS-I [10,11,21,22,23,24,25,26].

The RoB 2 and ROBINS-I tools were used for quality assessment [14,15]. The overall risk of bias in the included trials and study was considered “some concerns” for the RCT and “moderate” for non-randomized studies (Figure 5). The RCSs [11,12,23,24,25,26,27] were assigned as “moderate” risk bias due to confounding.

Rotman [8] was assessed as having not enough information for bias arising from the randomization process and some concerns for bias due to deviations from intended interventions. For the randomization process, the allocation sequence was insufficiently described, and there was no evidence to confirm that the sequence was adequately concealed. This lack of detail means it was not possible to determine whether the randomization was adequately implemented, resulting in a classification of not enough information. Regarding deviations from intended interventions, the nature of the intervention (radiotherapy versus observation) made blinding of participants, caregivers, and those delivering the intervention impossible. While objective outcomes such as recurrence and survival reduce the likelihood of bias, the potential influence on subjective assessments cannot be ruled out, leading to the classification of some concerns in this domain.

**Figure 6:**
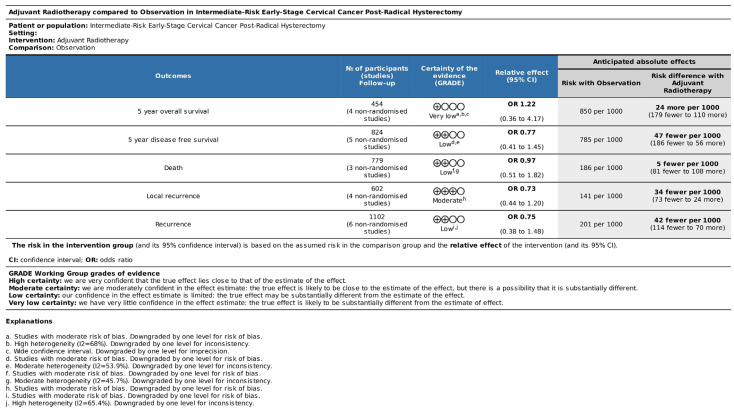
GRADE assessment.

According to the GRADE assessment [17], one outcome evaluated in this study was classified as moderate-quality evidence: local recurrence. Three outcomes had low-quality evidence: 5y-DFS, death, and recurrence. One outcome was classified as having very-low-quality evidence: 5y-OS. The main domains responsible for reducing the quality of evidence of the outcomes were risk of bias because of outcomes significantly carried out by studies with moderate risk of bias, imprecision due to wide confidence interval, and inconsistency of results because of heterogeneity. Quality assessment is detailed in Figure 6.

## 4. Discussion

In this systematic review and meta-analysis, nine studies were included, as well as one RCT and eight RCSs, with 1504 patients of whom 781 (52%) received adjuvant RT. We compared adjuvant RT versus no adjuvant treatment for patients with early-stage CC classified as intermediate risk according to GOG-92 [8]. Our findings suggest that adjuvant radiotherapy does not significantly reduce the risk of recurrence, local recurrence, death, 5y-OS, and 5y-DFS. However, TS ≥ 4 cm was an independent negative prognostic risk factor for recurrence.

A previous meta-analysis [27] of two RCTs [8,28] found a significantly lower risk of recurrence within 5 years and improved recurrence-free survival. These results were predominantly influenced by GOG-92, which contributed 91.5% and 93.2% of the weight, respectively. The inclusion criteria encompassed women with “early-stage” CC with intermediate risk, as well as those with positive pelvic lymph nodes (PLNs) and parametrial involvement. Although GOG-92 excluded such patients, Bilek’s [28] decision remains unclear. Bilek et al. [28] were not included in our meta-analysis because the full text was unavailable, probably due to its publication year. The divergent outcomes of the previous meta-analysis may be attributed to the GOG-92 [8] study’s significant influence.

The GOG-92 trial [8] demonstrated a significant reduction in recurrence and death in intermediate-risk early-stage CC treated with adjuvant RT. However, despite being non-randomized, other studies included in this meta-analysis did not significantly suggest a reduction in recurrence [10,11,21,23] or death [10,11]. Among the included studies, only Rotman [8] and Nie [24] favored adjuvant RT for recurrence, while Wang [23] supported it for 5y-DFS. The remaining studies did not show statistically significant differences in the outcomes of interest. The studies did not specify the interval between surgery and adjuvant treatment, and any potential impact of timing variability on the outcomes could not be assessed.

Several factors may help explain why more recent studies did not replicate the benefits observed in GOG-92. One important consideration is the methodological difference between studies. While GOG-92 was a randomized controlled trial, the contemporary studies in our analysis were retrospective cohort studies, which are more susceptible to bias, residual confounding, and variability in patient selection. Retrospective studies are subject to various limitations, including selection bias, missing data, inconsistencies in treatment application, and lack of standardized follow-up. These methodological differences can significantly impact the comparability of outcomes and may partly explain the observed differences in efficacy. Additionally, recent studies can benefit from advancements in imaging and staging, which allow for more accurate stratification of patients and exclusion of high-risk features prior to treatment decisions. Furthermore, surgical techniques have evolved, with greater consistency in the extent of radical hysterectomy and surgical expertise, which may independently reduce recurrence risk and lessen the need for adjuvant treatment.

It is important to highlight that the included studies did not evaluate adjuvant chemoradiotherapy (CRT). The CERVANTES trial [29] and GOG-263 (NCT01101451), which evaluates postoperative adjuvant RT or CRT for intermediate-risk early-stage CC, are not available and may offer new data from RT and CRT intervention. Nevertheless, Mahmoud [30] and Kim [31] reported no significant survival benefit for adjuvant CRT over adjuvant RT. Therefore, we suggest adjuvant RT or CRT should be considered with caution until the CERVANTES and GOG-263 trial results become available because adjuvant treatment may not offer additional benefits and could increase morbidity.

Our analysis indicates that DSI did not demonstrate prognostic significance as a risk factor for recurrence. Although the depth of stromal invasion influences FIGO staging, especially in early stages—and staging is directly associated with recurrence risk—our findings did not show a direct relationship between DSI and recurrence. Zhu et al. [32], however, reported that DSI is an important prognostic factor for OS and DFS in patients with early-stage CC. LVSI has been associated with poor prognostic outcomes, including decreased OS and DFS for early-stage CC [33]. Nonetheless, our analysis did not demonstrate a significant association between LVSI and recurrence risk. Additionally, data on DSI and LVSI were not uniformly reported across studies, with variations in definitions and assessment criteria. This inconsistency represents a methodological limitation that may have introduced variability in patient selection and outcome evaluation, potentially affecting the reliability of the pooled estimates.

The findings in this meta-analysis suggest that TS ≥ 4 cm was an independent negative prognostic risk factor for recurrence. The influence on staging and subsequent treatment options is further validated through recent changes in the FIGO staging system. Following the FIGO 2018 staging system, TS > 4 cm in diameter is now classified as stage IB3 or ≥IIA2, and the guidelines recommend primary treatment with CRT for these stages [6]. These current clinical recommendations, in accordance with our findings, emphasize the importance of tumor size in therapeutic decision-making and its impact on prognostic outcomes. The available data did not allow for a specific analysis of the impact of adjuvant RT on recurrence rates in patients with larger tumors. As such, conclusions regarding the efficacy of treatment in this subgroup remain limited.

A recent sub-analysis of the SCCAN study by Cibula et al. [34] found no significant benefit of adjuvant radiotherapy or chemoradiotherapy in intermediate-risk patients following radical surgery. The study, which included a large international cohort, demonstrated comparable 5y-DFS and OS between patients receiving adjuvant therapy and those managed with surgery alone. These results support the notion that adjuvant treatment may not confer additional survival advantage in this subgroup and highlight the importance of avoiding overtreatment.

Our study has some limitations. The meta-analysis revealed moderate to high heterogeneity in certain outcomes, such as recurrence and survival rates [11,23,24], which can be attributed to differences in study design, patient populations, and treatment protocols across the included studies. Although leave-one-out sensitivity analyses were performed and demonstrated consistent results after omitting each study, the underlying variability remains a concern. Furthermore, only nine studies were included in the analysis, most of which were retrospective cohort studies, increasing the risk of bias due to unmeasured confounding variables. Retrospective data collection can lead to inconsistencies in data quality and completeness. In summary, although the meta-analysis provides valuable insights into treatment outcomes for intermediate-risk early-stage cervical cancer, these limitations highlight the need for a cautious interpretation of the results. Future research should focus on larger, well-designed, prospective studies to address these limitations and provide more definitive conclusions.

## 5. Conclusions

The role of adjuvant RT for patients with intermediate-risk early-stage CC post-RH remains controversial and, based on our results, should be considered with caution until the CERVANTES and GOG-0263 trial results become available. The lack of significant improvement in recurrence rates and survival outcomes raises questions about the necessity of routine adjuvant RT for all intermediate-risk patients. This finding is particularly relevant given the potential side effects and quality of life implications associated with radiotherapy.

Moreover, this study emphasizes the need for personalized treatment strategies, considering factors such as tumor size and specific risk parameters to adapt interventions effectively. Methodological differences across studies, such as variations in study design, patient demographics, and treatment protocols, highlight the need for standardized approaches in future research to more accurately assess the efficacy of adjuvant RT.

## Figures and Tables

**Table 1:** Baseline characteristics of included studies.

Study	Year	Study Design	Country	FIGO Stage	No. of Patients	Age ^§^ (years)	Tumor Size ≥ 4 cm	+LSVI	DSI	No. of SCC Histology (%)	Follow-up (Months)
					adRT/RH	adRT/RH	adRT/RH	adRT/RH	adRT/RH	adRT/RH	
Rotman [8]	2006	RCT	USA	IB	137/140	NA	NA	NA	NA	103 (75.2)/ 115 (82.1)	120
Tuipae [21]	2012	RCS	Thailand	IB–IIA	18/97	NA	11/24	17/57	16/65	13 (72.2)/ 65 (67)	62.5
Akilli [10]	2020	RCS	Turkey	NA	68/66	54 ^†^/51 ^†^	28/17	57/42	62/58	53 (77.9)/ 55 (83.3)	61.05
Kim [22]	2020	RCS	South Korea	IB–IIA	53/30	51.6 ± 11.5/53.2 ± 14.2	38/21	35/15	48/17	48 (90.6)/ 21 (70.0)	40.4
Cao [11]	2020	RCS	China	IB1–IIA2	283/85	51 ^†^/47 ^†^	227/65	116/30	133/31	283 (100)/ 85 (100)	63
Wang [23]	2021	RCS	China	IB–IIA	21/52	50.7 (36–69)/52.4 (31–72)	8/6	16/45	NA	15 (71.4)/ 38 (73.1)	117.7
Nie [24]	2021	RCS	China	I–IIA	20/21	NA	NA	NA	NA	NA	62
Turkmen [25]	2022	RCS	Turkey	IB1–IIA2	67/116	52 (34–73) ^†^/ 50 (26–79) ^†^	25/37	46/60	NA	53 (37.6)/ 88 (62.4)	48
Tuscharoenporn [26]	2023	RCS	Thailand	IB–IIA	114/116	47.75 ± 10.03/ 46.22 ± 8.46	NA	103/104	0/0	81 (71.1)/ 79 (68.1)	76.1/95.4

**^§^** Mean (range) or mean ± SD; ^†^ median (range) or median ± SD. Abbreviations: adRT: adjuvant radiotherapy DSI: deep stromal invasion; FIGO: International Federation of Gynecology and Obstetrics; +LVSI: lymphovascular space invasion-positive; NA: not available; RCS: retrospective cohort study; RCT: randomized controlled trial; RH: radical hysterectomy; SCC: squamous cell carcinoma.

## Data Availability

All data relevant to the study are included in the article. Further information can be obtained from the corresponding author.

## Supplementary Materials

The following supporting information can be downloaded at: https://www.mdpi.com/article/10.3390/jcm14114002/s1, Table S1: Intermediate risk by GOG-92; Table S2: Search strategy; Table S3: Results from pooled analyses.

## Abbreviations

The following abbreviations are used in this manuscript:

CC	Cervical Cancer
CIs	Confidence Intervals
CRT	Chemoradiotherapy
DSI	Deep Stromal Invasion
FIGO	Federation of Gynecology and Obstetrics
GOG	Gynecologic Oncology Group
GRADE	Grading of Recommendation, Assessment, Development and Evaluations
HDI	Human Development Index
HR	Hazard Ratio
LVSI	Lymphovascular Space Invasion
NCCN	National Comprehensive Cancer Network
OR	Odds Ratio
PLN	Pelvic Lymph Nodes
PRISMA	Preferred Reporting Items for Systematic Reviews and Meta-Analysis
PROSPERO	Prospective Register of Systematic Reviews
RevMan	Review Manager
RCSs	Retrospective Cohort Studies
RCT	Randomized Clinical Trial
RH	Radical Hysterectomy
ROBINS-I	Risk of Bias In Non-randomized Studies of Interventions
ROBIVS	Risk of Bias VISualization
RoB 2	Risk of Bias 2
RT	Radiotherapy
TS	Tumor Size
WHO	World Health Organization
5y-DFS	5-year Disease-Free Survival
5y-OS	5-year Overall Survival
