# Supplementary material for: Adjuvant Radiotherapy for Intermediate-Risk Early-Stage Cervical Cancer Post Radical Hysterectomy: A Systematic Review and Meta-Analysis
Source: J Clin Med. 2025 Jun 5;14(11):4002. doi: 10.3390/jcm14114002 (PMC12155710; doi:10.3390/jcm14114002)
Supplement: Supplementary file 1 [file jcm-14-04002-s001.zip › jcm-3592898-supplementary.pdf]

Adjuvant Radiotherapy for Intermediate-Risk Early-Stage Cervical Cancer Post-Radical Hysterectomy: A Systematic Review and Meta-Analysis

Pedro Henrique Costa Matos da Silva, Gabriela Oliveira Gonçalves Molino, Maírla Marina Ferreira Dias, Ana Gabriela Alves Pereira, Nicole dos Santos Pimenta, Deivyd Vieira Silva Cavalcante, Ana Clara Felix de Farias Santos, Sarah Hasimyan Ferreira, Rodrigo da Silva Santos, Angela Adamski da Silva Reis.

Supplementary Material

Table S1. Intermediate risk by GOG-92

Table S2. Search strategy

Table S3. Results from pooled analyses

Table S1. Intermediate risk by GOG-92

**Sedlis, 1999 [8]**		
**LVSI**	**Stromal invasion**	**Tumor Size**
Positive (+)	Deep 1/3	any
Positive (+)	Middle 1/3	≥ 2cm
Positive (+)	Superficial 1/3	≥ 5cm
Negative (-)	Deep or middle 1/3	≥ 4cm
**Rotman, 2006 [9]**		
≥ 2 risk factors	DSI	
	LVSI	
	Tumor size ≥ 4cm	

DSI: deep stromal invasion; GOG: gynecological oncology group; LVSI: lymphovascular space involvement (LVSI)

Table S2. Search strategy

Data base	Search strategy	Results
PubMed	("uterine cervical neoplasms"[mh] OR "neoplasm, cervix" OR "cervix cancer" OR "cervical cancer") AND ("radiotherapy"[mh] OR "radiotherapy" OR "adjuvant pelvic radiation" OR "radiation treatments" OR "irradiation" OR "RT") AND ((("stromal invasion" OR "DSI" OR "stromal infiltration" OR "stromal") AND ("lymphovascular" OR "lymphovascular space" OR "lymphatic" OR "lymphatic space" OR "capillary lymphatic space" OR "LSVI" OR "CLS")) OR ("Sedlis" OR "Sedlis criteria" OR "GOG-92" OR "intermediate risk" OR "intermediate-risk"))	301*
Cochrane	("uterine cervical neoplasms"[mh] OR "neoplasm, cervix" OR "cervix cancer" OR "cervical cancer") AND ("radiotherapy"[mh] OR "radiotherapy" OR "adjuvant pelvic radiation" OR "radiation treatments" OR "irradiation" OR "RT") AND ((("stromal invasion" OR "DSI" OR "stromal infiltration" OR "stromal") AND ("lymphovascular" OR "lymphovascular space" OR "lymphatic" OR "lymphatic space" OR "capillary lymphatic space" OR "LSVI" OR "CLS")) OR ("Sedlis" OR "Sedlis criteria" OR "GOG-92" OR "intermediate risk" OR "intermediate-risk"))	41*
EMBASE	('uterine cervical neoplasms'/exp OR 'uterine cervical neoplasms' OR 'neoplasm, cervix' OR 'cervix cancer'/exp OR 'cervix cancer' OR 'cervical cancer'/exp OR 'cervical cancer') AND ('radiotherapy'/exp OR 'radiotherapy' OR 'adjuvant pelvic radiation' OR 'radiation treatments' OR 'irradiation'/exp OR 'irradiation' OR 'rt'/exp OR 'rt') AND (('stromal invasion'/exp OR 'stromal invasion' OR 'dsi' OR 'stromal infiltration' OR 'stromal') AND ('lymphovascular'/exp OR 'lymphovascular' OR 'lymphovascular space' OR 'lymphatic'/exp OR 'lymphatic' OR 'lymphatic space' OR 'capillary lymphatic space' OR 'lsvi' OR 'cls'/exp OR 'cls') OR 'sedlis' OR 'sedlis criteria' OR 'gog-92' OR 'intermediate risk' OR 'intermediate-risk')	617*

* Search strategy performed in August 2024

Table S3. Results from pooled analyses

Outcome	Included studies, (n)	No. of patients RH + RT / RH	Effect estimate (95% CI)	P-value	I², %
Death	3	488 / 291	OR 0.97 (0.52 – 1.80)	0.91	46
Local recurrence	4	275 / 327	OR 0.73 (0.44 – 1.20)	0.22	0
Recurrence	6	619 / 483	OR 0.75 (0.38 – 1.46)	0.39	66
5y-DFS	5	457 / 367	OR 0.78 (0.42 – 1.43)	0.42	54
5y-OS	4	168 / 286	OR 1.22 (0.36 – 4.18)	0.75	68

CI: confidence interval; OR: odds ratio; RH: Radical Hysterectomy; RT: Radiotherapy
